# Pretargeted brain PET imaging reveals amyloid-β pathology using a TCO-modified antibody and a fluorine-18-labeled tetrazine

**DOI:** 10.1186/s40035-025-00532-2

**Published:** 2025-12-26

**Authors:** Sara Lopes van den Broek, Jonas Eriksson, Qiaojun Yang, Nadja M. Bucher, Eva Schlein, Lorenzo J. I. Balestri, Luke R. Odell, Dag Sehlin, Stina Syvänen

**Affiliations:** 1 Department of Public Health and Caring Sciences, Uppsala University, 751 85 Uppsala, Sweden; 2 Department of Medicinal Chemistry, Uppsala University, 751 85 Uppsala, Sweden; 3 PET Centre, Uppsala University Hospital, 751 85 Uppsala, Sweden

**Keywords:** Pretargeted PET imaging, Blood–brain barrier, Bispecific antibody, Amyloid-β, Fluorine-18 tetrazine

## Abstract

**Background:**

Antibody-based positron emission tomography (PET) imaging holds great promise for visualizing disease-related proteins in the brain. However, its clinical utility is limited by poor antibody penetration across the blood–brain barrier (BBB) and the requirement for long-lived radionuclides due to slow antibody pharmacokinetics. Pretargeted imaging strategies, in which antibody administration and radioligand injection are separated in time, enable the use of short-lived, high-resolution PET-compatible radionuclides such as fluorine-18.

**Methods:**

A bispecific antibody, Bapi-Fab8D3, which targets both amyloid beta (Aβ) and the transferrin receptor (TfR) for TfR-mediated transport across the BBB, was conjugated with trans-cyclooctene (TCO) to enable in vivo click chemistry. Following antibody administration to Alzheimer's disease (AD) model mice and sufficient time for accumulation at intrabrain Aβ deposits, a fluorine-18-labeled tetrazine was injected to react in vivo with the TCO handles on the antibody. PET imaging, autoradiography, ex vivo quantification, and histological analyses were performed to evaluate the specificity and distribution of the imaging signal.

**Results:**

Bapi-Fab8D3 retained its binding affinity for both Aβ and TfR after TCO-conjugation. In brain sections, reactive TCOs were detected up to three days after antibody injection, indicating successful transcytosis across the BBB and stable target engagement. Pretargeted PET imaging after fluorine-18-labeled tetrazine injection revealed significantly higher signals in AD mice that received TCO-Bapi-Fab8D3 compared to wild-type controls or AD mice that received the unmodified antibody. The uptake pattern corresponded to Aβ plaque distribution, and quantitative analysis showed increased signal in AD-relevant brain regions including the hippocampus and thalamus.

**Conclusions:**

This study demonstrates successful pretargeted PET imaging of brain Aβ pathology using a systemically administered bispecific antibody capable of BBB penetration and a fluorine-18-labeled tetrazine. These findings establish a generalizable strategy for high-contrast in vivo imaging of brain protein targets using pretargeted PET, with the potential to expand molecular imaging to protein targets in the brain that are currently inaccessible.

**Supplementary Information:**

The online version contains supplementary material available at 10.1186/s40035-025-00532-2.

## Background

With an ageing population, the prevalence of neurodegenerative diseases is expected to rise significantly. Currently, treatment options remain limited, with most available drugs targeting symptoms rather than the underlying causes of these diseases. However, a promising therapeutic strategy that has emerged over the past decades is immunotherapy [1, 2]. Notably, the recently approved antibody lecanemab targets amyloid-β (Aβ) in the brains of Alzheimer’s disease (AD) patients, facilitating its clearance. The approval of lecanemab (NCT03887455), and the ongoing clinical evaluations (NCT04437511, NCT02484547, NCT04639050) of similar antibodies expected to gain approval in the coming years, have been greatly supported by positron emission tomography (PET) imaging of aggregated Aβ in the brain. PET imaging has convincingly demonstrated that these antibodies reduce brain levels of insoluble Aβ, i.e., plaques [3, 4]. Despite its value, PET imaging of aggregated proteins is currently limited to detecting amyloids, the characteristic β-sheet structure found in deposits of densely packed Aβ fibrils [5, 6]. It is important to note that amyloid-PET is not specific to the Aβ protein, as other proteins can also form amyloid structures. Moreover, antibodies such as lecanemab target a range of soluble and diffuse Aβ aggregates, rather than the amyloid core of plaques. This creates a partial mismatch between the therapeutic target of immunotherapy and the structures visualized using amyloid PET imaging. For other neurodegenerative diseases characterized by pathological protein aggregation, such as those involving alpha-synuclein, TAR DNA-binding protein 43, or proteins related to neuroinflammation, there are currently either suboptimal or no effective PET radioligands available. Developing small molecule-based PET ligands for these proteins has proven challenging due to off-target binding and poor imaging contrast [7–10].

An alternative approach to small molecule-based PET ligands involves radiolabeling antibodies or antibody fragments for use as PET radioligands. Antibodies offer high target specificity, but suffer from poor brain delivery and long circulation times, which limit their use as radioligands in PET imaging. To overcome the low brain delivery, bispecific antibody formats have been engineered by fusing a transferrin receptor (TfR)-binding domain to the primary antibody. The TfR serves as a shuttle, significantly enhancing antibody transport across the blood–brain barrier (BBB) [11–15]. Nonetheless, the long systemic half-life of antibodies remains a significant hurdle for antibody-based PET, as it necessitates imaging several days after administration to allow clearance of background signal from circulating radiolabeled antibodies. Additionally, it requires the use of long-lived radionuclides, such as iodine-124 or zirconium-89, which results in relatively high radiation doses to patients. Clinically, it would be preferable to use fluorine-18 (^18^F), a radionuclide with a half-life of just under two hours.

**Fig. 1:**
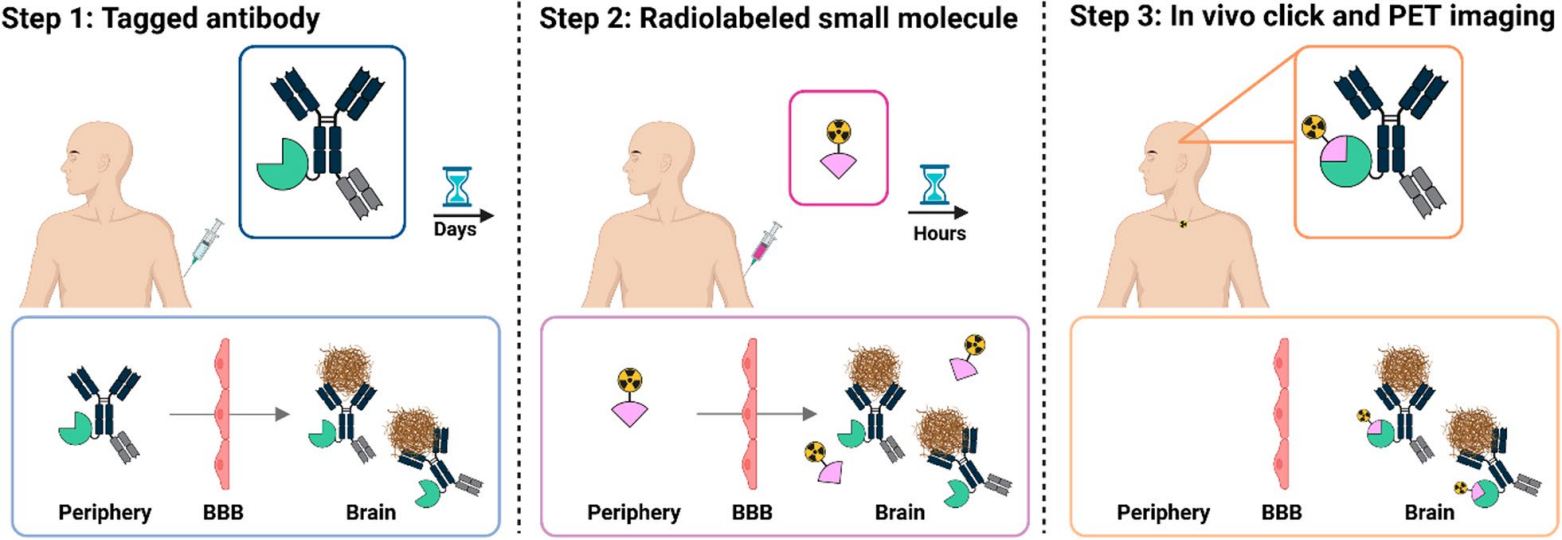
Overview of pretargeted PET imaging with brain-penetrant antibodies. In pretargeted PET imaging using bispecific brain-penetrant antibodies, Step 1 involves modifying the antibody with a reactive “handle” prior to administration. After injection, the antibody crosses the blood–brain barrier (BBB), binds to its target protein (in this study amyloid-β), and is given time to clear from the circulation. Step 2 consists of administering a small molecule carrying the radionuclide (in this study fluorine-18) and a complementary reactive group. This radiolabeled molecule rapidly reacts with the antibody’s handle at the target site, thereby labelling the target protein in vivo. Step 3 occurs once the unbound radiolabeled molecule has cleared, at which point PET imaging is performed to visualize the labeled target. Illustrations were created with BioRender.com

In addition to its short half-life, ^18^F emits positrons with low kinetic energy and has a high positron emission fraction, which contribute to high-resolution images and lower radiation burden. To enable antibody-based PET imaging with ^18^F, the pretargeted imaging concept has been proposed (Fig. 1) [16–19]. In this strategy, the antibody is modified with a reactive “handle” (e.g., trans-cyclooctene, TCO) but is not radiolabeled prior to administration. After administration, the antibody binds to its target protein and is given time to clear from circulation without concerns related to radioactive decay or patient radiation dose. At the optimal time point, defined by high target engagement and low systemic antibody levels, a small molecule carrying the radionuclide and a complementary reactive group (e.g., a tetrazine) is administered. This radiolabeled molecule rapidly reacts with the antibody’s handle at the target site, effectively labelling the target protein of interest in vivo [19–21].

In this study, we establish a robust and versatile platform for pretargeted brain PET imaging by combining TCO-modified bispecific antibodies capable of TfR-mediated brain delivery with ^18^F-labeled tetrazines for rapid in vivo click chemistry. By leveraging the high specificity and efficiency of the tetrazine ligation reaction, we demonstrate specific visualization of brain Aβ pathology. Importantly, while this work focuses on Aβ, the strategy can be readily adapted to a wide range of brain targets, enabling the imaging of diverse pathologies in both research and clinical settings, provided that a suitable antibody is available.

## Materials and methods

### Animals

All ex vivo biodistribution and PET imaging studies were performed in old (12–19 months) in-house-bred App^NL-G-F^ knock-in mice, expressing mouse amyloid precursor protein (*APP*) with a humanized Aβ sequence as well as the Swedish (KM670/671NL), Arctic (E693G) and Beyreuter/Iberian (I716F) mutations, leading to rapid pathology progression [22]. Age-matched wild-type (WT) mice (C57BL/6JBomTac) were used as a control. Ex vivo pretargeting experiments also included in-house-bred Tg-ArcSwe mice, a transgenic model that harbors the Swedish and the Arctic APP mutations and is characterized by high levels of aggregated Aβ that forms dense-core plaques [23, 24]. To study brain distribution of an assumingly non-brain penetrant tetrazine (later used for blocking peripheral TCO groups), 4-month-old WT mice were used. Both males and females were included in all studies (Table S1). The mice were housed in an approved animal facility at Uppsala University with ad libitum access to food and water. All described procedures were approved by the Uppsala Country Animal Ethics board (5.8.18–16493/2024) following the legislation and regulations of the Swedish Animal Welfare Agency and European Communities Council Directive of 22 September 2010 (2010/63/EU).

### Antibody modifications

The bispecific antibody Bapi-Fab8D3 is derived from the full-length IgG antibody bapineuzumab (Bapi), which targets the N-terminus of Aβ and has previously been investigated as an anti-Aβ therapeutic [25]. An Fab fragment of the murine TfR-binding antibody 8D3 (Fab8D3) was fused to the C-terminus of one of the Bapi heavy chains, resulting in a bispecific format. Mutations (L234A, L235A, P329G) were introduced to the Fc-domain to reduce effector functions [26, 27]. This design closely resembles the BrainShuttle format developed by Roche [28, 29]. Bapi-Fab8D3 was transiently expressed in Expi293F cells (ThermoFisher, Waltham, MA, A14527) following a previously described protocol [30].

Bapi-Fab8D3 was then modified at lysine residues using TCO-NHS ester, following a previously described procedure [16]. Briefly, sodium carbonate buffer (1 M, pH 8.0) was added to phosphate-buffered saline (PBS) containing Bapi-Fab8D3 (1.5–3.0 mg/mL) to achieve a final buffer concentration of 30 mM. Axial TCO-NHS ester (BioNordika, Solna, Sweden, VEC-CCT-1509-25) dissolved in dimethylsulfoxide (DMSO) was then added at a 20–50 molar excess relative to the antibody. The reaction mixture was incubated at 600 rpm for 2 h at room temperature in the dark. Unreacted TCO-NHS ester was subsequently removed using Zeba Spin desalting columns (7 K MWCO, 0.5 mL; ThermoFisher, 89882), and the modified antibody was eluted in PBS (pH 7.4). The final protein concentration was determined using a spectrophotometer (DS-11, DeNovix, Wilmington, DE).

Enzyme-linked immunosorbent assays (ELISA) was used to investigate antibody binding towards Aβ and murine TfR. The 96-well half-area plates (Corning Inc., New York, NY) were coated overnight at 4 °C with Aβ (50 nM diluted in PBS), murine TfR (50 µL/well, 5 µg/mL diluted in PBS), or anti-human IgG (hIgG) (0.5 µL/well, 5 µg/mL diluted in PBS). The wells were blocked with 1% bovine serum albumin (BSA) in PBS for 1 h. The antibodies Bapi-Fab8D3 and TCO-Bapi-Fab8D3 variants were serially diluted in incubation buffer (PBS with 0.1% BSA, 0.05% Tween-20 and 0.15% Kathon) from 50 nM to 3.2 pM and incubated for 2 h. The plates were washed with wash buffer and detected with horseradish peroxidase (HRP)-coupled goat hIgG-F(ab’)2 (1:2000, Jackson ImmunoResearch Laboratories, West Grove, PA, 109-036-006). Plates were developed with K blue aqueous TMB substrate (Neogen Corp., Lexington, KY), quenched with 1 M H_2_SO_4_ and read with a spectrophotometer at 450 nm. The data were corrected for concentration obtained from the hIgG ELISA.

The number of TCOs conjugated per antibody was determined by SDS-PAGE. Aliquots of TCO-Bapi-Fab8D3 in PBS (10 μL, 0.005–0.025 nmol expected TCO per sample) were mixed with aliquots of CF647-Tetrazine (Bionordika, Solna, Sweden, BIT-96056, 5 µL, 0.05 nmol) in PBS containing 10% DMSO, ensuring an excess of tetrazine in comparison to TCOs. The samples were mixed and incubated at 600 rpm for 1 h at 37 °C. Bolt Sample Buffer (5 μL, B0007, Invitrogen, Carlsbad, CA) was added to each sample and the samples were heated to 95 °C and incubated at 600 rpm for 2 min. Samples were applied onto SDS-PAGE gels (Bolt Bis–Tris Plus Mini Protein Gels, 4%–12%, 1.0 mm, 12-well, NW04122BOX, Invitrogen), alongside a molecular weight standard (PageRuler Plus Prestained Protein Ladder, 26619, Invitrogen) and a CF647-Tetrazine control followed by electrophoresis in Bolt MES SDS running buffer (B0002, Invitrogen) for 20 min at 200 V. After electrophoresis, gels were removed from their cassette and directly imaged for AF647-fluorescent intensity using an iBright FL1500 Imaging System (Invitrogen) and fluorescence was quantified according to previously published procedures [16, 17]. After fluorescence quantification, which reflects the number of TCO groups conjugated per antibody based on tetrazine binding, the gel was stained with InstantBlue Coomassie Protein Stain (ab119211, Abcam, Cambridge, UK), and an image was taken with the iBright FL1500 Imaging System.

### Radiochemistry

#### Synthesis of [^68^ Ga]Ga-DOTA-PEG_11_-Tz

To investigate the reactivity of TCO groups after in vivo accumulation in the brain, the polar tetrazine DOTA-PEG_11_-Tz (Fig. S1a) was selected for incubation with brain sections prepared from mice administered with TCO-Bapi-Fab8D3. This tetrazine was radiolabeled with gallium-68 (^68^Ga) to enable detection by autoradiography, following the protocol described below.

[^68^Ga]GaCl_3_ (329 MBq) in 0.1 M HCl (1.3 mL) was added to a reaction vial containing DOTA-PEG_11_-Tz (50–100 µg, 0.04–0.08 µmol) in ammonium acetate buffer (0.2 M, pH 7, 40 µL), 70% ethanol (400 µL), and additional ammonium acetate buffer (0.2 M, pH 6, 1000 µL). The reaction mixture was heated at 90 °C for 10 min, then purified using a solid-phase extraction (SPE) cartridge (Sep-Pak Plus Light C18, WAT036805, Waters, Milford, MA), which had been pre-conditioned with 70% ethanol (2.5 mL) and water (5 mL). The SPE cartridge was washed with water (5 mL), and the product was eluted with ethanol (1 mL), yielding [^68^Ga]Ga-DOTA-PEG_11_-Tz with a decay-corrected radiochemical yield (RCY) of 66% corresponding to 179 MBq (non-decay-corrected RCY of 54%). Radiochemical purity (RCP) was assessed by radio-thin layer chromatography (radio-TLC) using silica gel 60 plates (1.05554.0001, Merck) and citrate buffer (0.1 M, pH 4) as the mobile phase. The retention factors (Rf) were: [^68^Ga]Ga-DOTA-PEG_11_-Tz: Rf = 0; free [^68^Ga]GaCl_3_: Rf = 1. The RCP was > 98%, and the molar activity (Am) was 5.7 GBq/µmol.

#### Synthesis of ^18^F-labeled tetrazines

For in vivo pretargeted imaging, two brain-penetrant tetrazines were used, [^18^F]HTzA and [^18^F]MeTzA (Fig. S1b, c). The labeled tetrazines were synthesized as previously described [31, 32], providing [^18^F]MeTzA with a Am of 109 ± 5 GBq/μmol, a radioactivity yield of 1380 ± 260 MBq, and a RCP of 97.5% ± 1.5% (*n* = 4). [^18^F]HTzA was synthesized accordingly, affording the labeled tetrazine with a Am of 123 ± 34 GBq/μmol, a radioactivity yield of 830 ± 150 MBq, and a RCP of 98.0% ± 1.0% (*n* = 5).

### Ex vivo* pretargeted imaging*

App^NL-G-F^, Tg-ArcSwe and WT mice (*n* = 6 in total, *n* = 1 per group) were intravenously (i.v.) injected with 20 nmol/kg Bapi-Fab8D3 or TCO-Bapi-Fab8D3 via the tail vein. This dose was selected based on previous studies, with the aim of achieving high brain concentrations without blocking TfR-mediated transcytosis [33]. Three days later, mice were anesthetized with isoflurane, followed by transcardial perfusion with 0.9% NaCl to remove blood. In parallel, App^NL-G-F^, Tg-ArcSwe and WT mice (*n* = 3 in total, *n* = 1 per group) that did not receive antibody were perfused as additional controls. Brains were isolated and divided into the left and right hemispheres, and immediately frozen. Sagittal brain sections (20 μm, *n* = 6 per brain) between 1.2 and 2.2 mm lateral to the midline were prepared using a Cryostar NX70 (ThermoFischer). The sections were then incubated with [^68^Ga]Ga-DOTA-PEG_11_-Tz (20 nM in PBS-0.1% Tween20) for 45 min at room temperature. Afterwards, sections were washed in cold PBS (3 × 3 min) and cold water (1 × 1 min). After drying, sections were exposed to a phosphor imaging plate (BAS-IP SR 2040, Fujifilm, 28956477) for 2.5 h and the plate was scanned in an Amersham Typhoon IP phosphor imager (GE Healthcare, 29187194) at a resolution of 50 μm. To visualize the distribution of Aβ pathology, brain sections from separate App^NL-G-F^, Tg-ArcSwe and WT mice were stained with Luminescent Conjugated Oligothiophene (LCO) HS-84 according to a previously published procedure [34].

### PET imaging

#### Selection of [^18^F]tetrazine

To investigate and compare the pharmacokinetics of the two tetrazines, [^18^F]HTzA (10.4 ± 4.4 MBq) and [^18^F]MeTzA (12.9 ± 4.5 MBq) were intravenously injected into App^NL-G-F^ and WT mice. A 90-min PET scan (Mediso NanoPET/MR, Mediso Medical Imaging System, Hungary) was initiated at the time of injection, followed by a 5-min computed tomography (CT) scan (Mediso NanoSPECT/CT, Mediso Medical Imaging System). Anesthesia was maintained throughout the scanning procedure using 3.5%–4.0% sevoflurane in a 0.5 L/min flow of 50% oxygen and 50% medical air. After the CT scan, the animals were returned to their home cages. At 3.5 h post-radioligand administration, the mice were again anesthetized and subjected to a 60-min PET scan, followed by an additional CT scan. At 5 h post-injection, a terminal blood sample was obtained from the heart before the mice were perfused, and the brain and peripheral organs were collected. Radioactivity in the tissues was quantified via γ-counting using a 2480 Wizard automatic gamma counter (PerkinElmer). PET data were reconstructed on a 160 × 160 × 128 grid with voxel dimensions of 0.5 × 0.5 × 0.6 mm^3^ using three-dimensional ordered-subsets expectation maximization (20 iterations). CT data were reconstructed using filtered back-projection. PET and CT images were processed using the PMOD image analysis software (PMOD Technologies, Zurich, Switzerland, version 4.105). PET images were co-registered to the corresponding CT scans, and the CT images were manually aligned to a standard brain atlas using the Fuse module. Volumes of interest (VOIs) were defined based on this alignment and applied to the dynamic PET data.

All measured activity concentrations, obtained either by PET imaging or γ-counting, were converted to standardized uptake values (SUV). SUV is a dose- and body weight-normalized measure of radioligand concentration that enables comparison between subjects administered with different amounts of radioactivity and between individuals with varying body weights. SUV was calculated as follows: SUV = Tissue radioactivity concentration (Bq/mL) / Injected activity (Bq) per body weight (g).

#### In vivo brain delivery of [^68^Ga]Ga-DOTA-PEG_11_-Tz

DOTA-PEG_11_-Tz*,* previously used to determine reactivity of TCO groups on brains sections, was also investigated as an agent to block TCO groups on TCO-Bapi-Fab8D3 present in the circulation to avoid them reacting with the brain penetrant [^18^F]tetrazines used for pretargeting. For this, it was crucial to ensure that the DOTA-PEG_11_-Tz did not enter the brain as this could reduce the number of available TCO for the pretargeted PET. Hence, 4-month-old WT mice received i.v. (*n* = 6) or intraperitoneal (i.p.) (*n* = 3) injection of 5.6 ± 1.8 MBq [^68^Ga]Ga-DOTA-PEG_11_-Tz. The i.v. administered animals were euthanized after perfusion at 3 min or 6 h, while the i.p. administered animals were euthanized at 6 h. Radioactivity in the brain was quantified via γ-counting.

#### Pretargeted imaging

To evaluate pretargeted imaging, mice received either Bapi-Fab8D3 or TCO-Bapi-Fab8D3 at a dose of 20 nmol/kg three days before PET scanning. On the day of scanning, the non–brain-penetrant tetrazine DOTA-PEG_11_-Tz was administered i.p. at a dose of 100 nmol/kg to block TCO groups on TCO-Bapi-Fab8D3 present in the blood circulation. This dose corresponded to a fivefold molar excess relative to the administered TCO dose, and likely represented approximately a 100-fold molar excess compared to the amount of TCOs remaining in circulation three days after TCO-Bapi-Fab8D3 injection. Two hours later, the animals were positioned in the PET scanner, injected with 13.2 ± 3.7 MBq of [^18^F]HTzA, and scanned for 90 min, followed by a 5-min CT scan. As in the tetrazine-only protocol, the mice were returned to their home cages and rescanned at 3.5 h post-radioligand administration. A subset of animals did not undergo the initial 90-min scan but were scanned only at the 3.5-h time point. These mice remained anesthetized during the first 90 min to match the experimental conditions of the group that also received an early scan. Brain, terminal blood and organ isolation, as well as image post-processing, were conducted as described in the previous section.

### Quantification of brain Aβ

The cerebrum of the left hemisphere isolated from PET-scanned animals was homogenized with a Precellys Evolution (Bertin Technologies, Montigny-le-Bretonneux, France) (4 × 10 s at 5500 rpm) at a 1:5 weight/volume ratio in Tris-buffered saline (TBS) with 1% complete protease inhibitor (Sigma). After centrifugation for 1 h at 16,000 × *g*, the supernatant was immediately removed and frozen. Pellets were dissolved in 70% formic acid (FA) and centrifuged at 16,000 × *g* for 1 h followed by supernatant collection. Concentrations of Aβ38, Aβ40, and Aβ42 in FA-extracted brain homogenate was quantified using the V-PLEX® Aβ peptide panel 1 (6E10) immunoassay (Meso Scale Discovery, Rockville, MD, K15200E). Samples were neutralized with 2 M Tris and diluted in assay diluent (1:10,000) before being loaded in duplicate onto pre-coated and blocked 96-well plates together with the secondary Aβ antibody 6E10 conjugated to a SULFO-TAG for electro-chemiluminescent detection. After 2 h of incubation, plates were washed with PBS containing 0.05% Tween, and Meso Scale Discovery read buffer was added. Plates were read with a MESO QuickPlex SQ instrument (Meso Scale Discovery).

### Statistics

Data are presented mean ± standard deviation (SD). One-way ANOVA followed by Tukey’s multiple comparisons test or two-way ANOVA followed by Šídák’s multiple comparisons test was used to correct for multiple comparisons. All tests were two-tailed, with a significance level set at 95%. *P* < 0.05 was considered as statistically significant. Graphs and statistical analyses were performed using GraphPad Prism version 10.4.2 (GraphPad Software, San Diego, CA).

## Results

### Affinity is preserved after TCO-modification

**Fig. 2:**
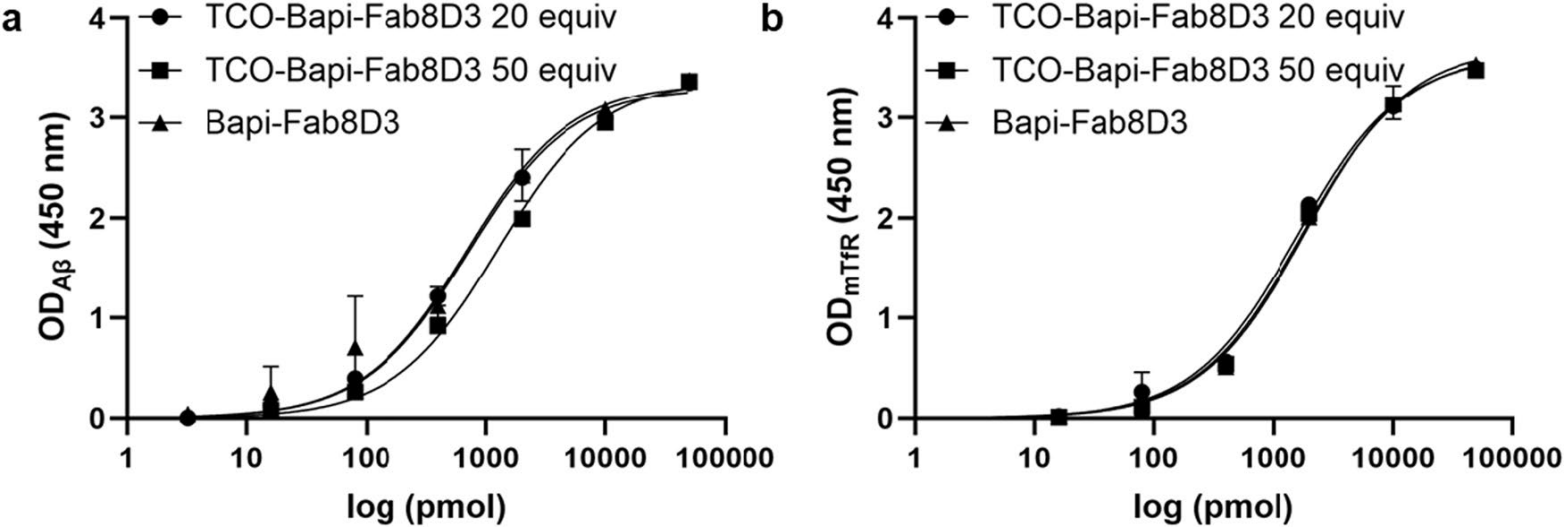
ELISA results of Bapi-Fab8D3 with TCO modification.** a** Binding affinity toward amyloid-β (Aβ) following modification of Bapi-Fab8D3 with 20 and 50 TCO equivalents, compared to the unmodified antibody, showed retained affinity relative to the unmodified antibody. **b** Binding affinity toward the transferrin receptor (TfR) after modification with 20 and 50 TCO equivalents demonstrated preserved affinity with both modifications relative to the unmodified antibody

The bispecific antibody Bapi-Fab8D3, in which the Bapi domain targets Aβ and the Fab8D3 domain binds to the murine TfR, was modified with varying molar excess of TCO over antibody. Modification with 20 and 50 molar equivalents of TCO resulted in the conjugation of an approximate average of 6 and 9 TCO moieties per Bapi-Fab8D3 molecule, respectively. Affinity towards both targets, Aβ and TfR, was preserved with both modifications (Fig. 2a, b). Therefore, the Bapi-Fab8D3 modified with 50 equivalents of TCO was selected for subsequent experiments.

### TCO-Bapi-Fab8D3 accumulates at Aβ aggregates with reactive TCOs at 3 days post administration

**Fig. 3:**
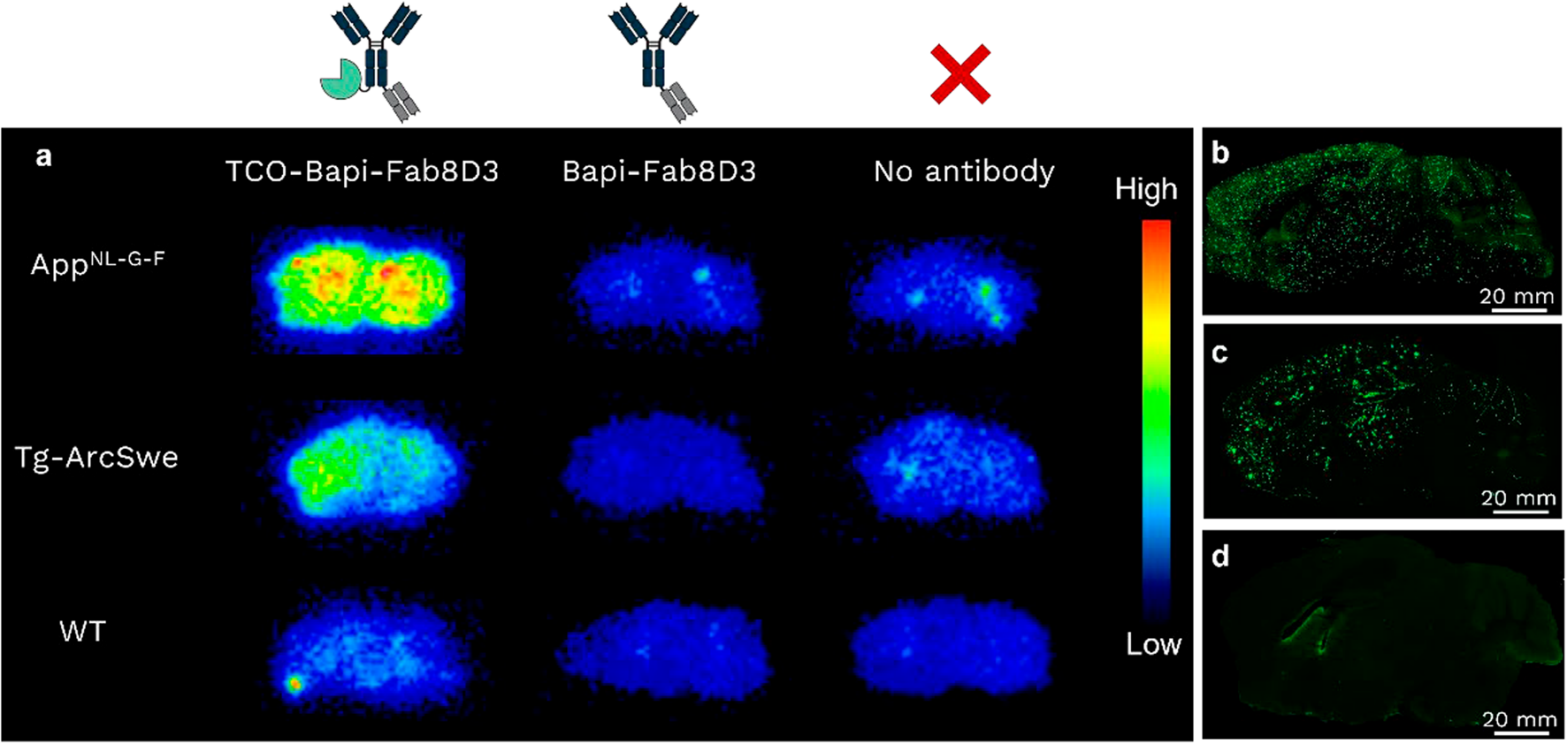
TCO groups remain reactive in vivo, enabling selective signal localization to pathology-rich regions. **a** Autoradiography images of brain sections prepared from mice administered with TCO-Bapi-Fab8D3, Bapi-Fab8D3, or no antibody, after incubation with a gallium-68 radiolabeled tetrazine. Only sections prepared from mice with Aβ pathology (App^NL-G-F^ and Tg-ArcSwe) show retention of the tetrazine, indicating that these sections contained reactive TCO groups attached to Aβ-bound antibody. Mice receiving unmodified Bapi-Fab8D3 or no antibody, as well as wild-type (WT) control mice without Aβ pathology showed no signal. TCO, trans-cyclooctene. **b–d** Representative images of LCO-stained Aβ pathology in App^NL-G-F^(**b**), Tg-ArcSwe (**c**) and WT (**d**) sagittal brain sections, respectively

To assess the in vivo reactivity of the TCO groups, TCO-Bapi-Fab8D3 was administered to two mouse models expressing Aβ pathology (App^NL-G-F^ and Tg-ArcSwe), as well as to WT control mice in an exploratory, qualitative experiment. For comparison, the unmodified Bapi-Fab8D3 was also administered. Three days post-injection, brains were collected and sagittal sections were prepared from the right hemisphere. As additional controls, brain sections were also obtained from mice that had not received any antibody. After incubation with the highly polar and reactive [^68^Ga]Ga-DOTA-PEG_11_-Tz, the sections were subjected to autoradiography. Sections from App^NL-G-F^ and Tg-ArcSwe mice that had received TCO-Bapi-Fab8D3 exhibited a clear autoradiographic signal, indicating that the antibody successfully crossed the BBB, bound to Aβ aggregates, and crucially, that TCO groups remained reactive after in vivo administration and target binding (Fig. 3a). In contrast, no specific signal was observed in sections from WT mice injected with TCO-Bapi-Fab8D3, mice injected with unmodified Bapi-Fab8D3, or non-injected controls (Fig. 3a). The spatial distribution of the signals detected by autoradiography in App^NL-G-F^ and Tg-ArcSwe mice that had received TCO-Bapi-Fab8D3 injection corresponded to brain regions typically containing high levels of Aβ [35, 36] as shown by LCO staining of Aβ deposits (Fig. 3b–d). While the App^NL-G-F^ mice exhibited widespread Aβ pathology, the Tg-ArcSwe mice displayed somewhat less extensive pathology, with particularly low levels in the cerebellum, a region that is less affected in this model.

### Brain pharmacokinetics and systemic elimination of two ^18^F-labeled tetrazines

**Fig. 4:**
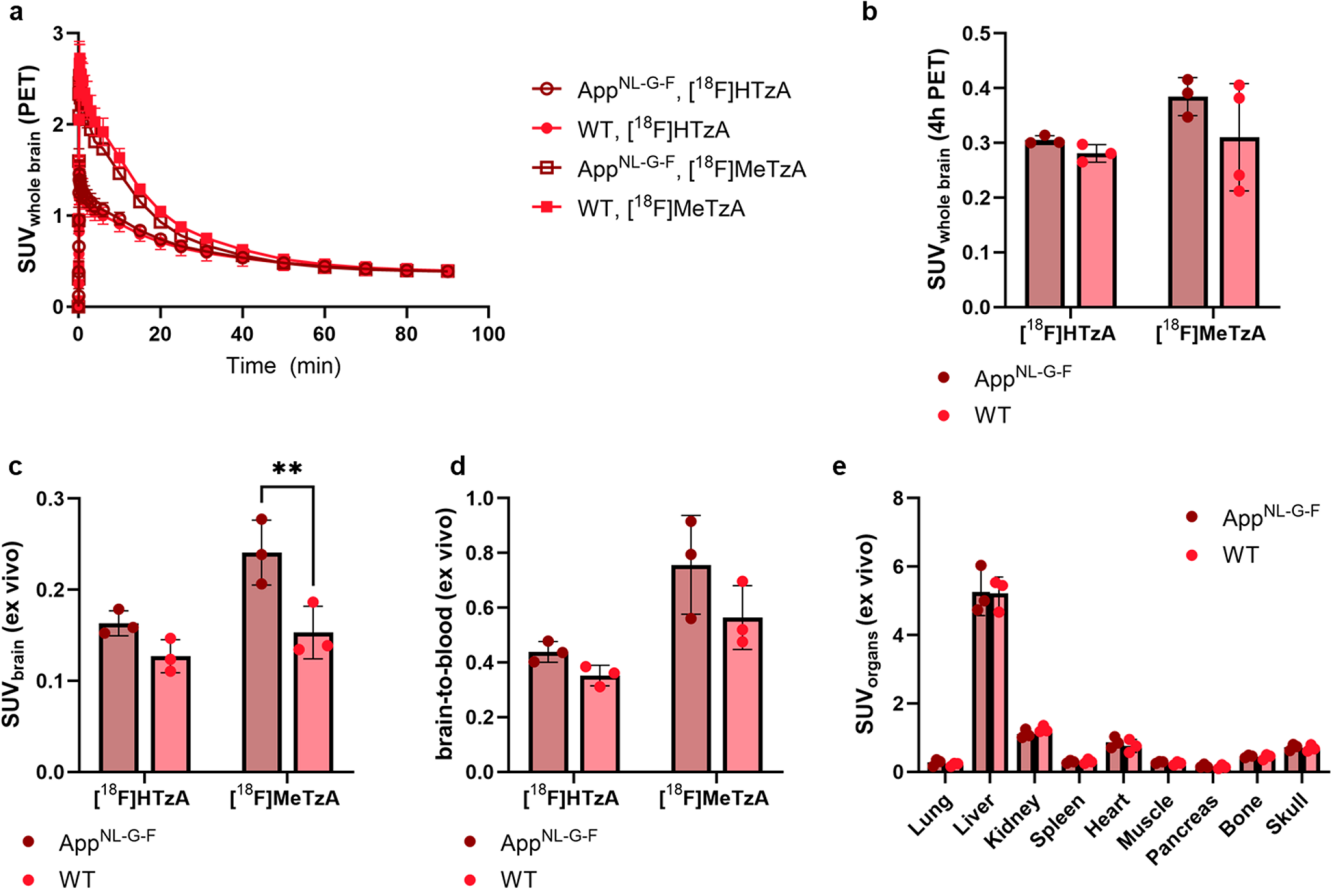
Brain concentrations of two fluorine-18 (^18^F)-tetrazines. **a** In vivo brain concentrations of [^18^F]HTzA and [^18^F]MeTzA, expressed as SUV, in App^NL-G-F^ and wild-type (WT) mice over the 90-min scanning period. **b** In vivo brain concentrations, expressed as SUV, at 4 h post administration of [^18^F]HTzA or [^18^F]MeTzA in App^NL-G-F^ and WT mice. **c** Ex vivo measured concentrations, expressed as SUV at 5 h post administration, in perfused isolated brain after PET scanning in App^NL-G-F^ and WT mice. **d** Ex vivo brain-to-blood concentration ratio at 5 h post administration in App^NL-G-F^ and WT mice. **e** Ex vivo concentrations in selected peripheral organs at 5 h post administration of [^18^F]HTzA in App^NL-G-F^ and WT mice. Mean ± SD of *n* = 3 for HTzA per genotype and *n* = 2 for MeTzA per genotype (**a**); mean ± SD of *n* = 3 per condition, except for WT [^18^F]MeTzA where *n* = 4 (**b**); mean ± SD of *n* = 3 per condition (**c–e**). **P < 0.01, two-way ANOVA Sidak’s multiple comparison (**b–e**). SUV, standardized uptake value, a dose- and weight-normalized concentration

For in vivo imaging using the pretargeted approach, the radiolabeled tetrazine should ideally enter the brain rapidly, and unbound (non-reacted) tetrazine should display efficient clearance from the brain and be eliminated from the blood quickly to allow for high imaging contrast. Two tetrazines, MeTzA and HTzA, radiolabeled with the clinically preferred radionuclide ^18^F, were evaluated in App^NL-G-F^ and WT mice. Both tetrazines exhibited rapid brain entry, reaching peak concentrations within 5 min post administration. The [^18^F]MeTzA showed higher peak brain uptake than [^18^F]HTzA (Fig. 4a). At 4 h post-injection, brain concentrations measured in vivo by PET were similar for both tetrazines, with no significant differences between App^NL-G-F^ and WT mice and with improved clearance compared to earlier time points (i.e., 1.5 h) (Fig. 4b, Fig. S2). However, in saline-perfused brains, isolated after the PET scan, App^NL-G-F^ mice displayed higher ex vivo concentrations of [^18^F]MeTzA compared to WT controls (Fig. 4c). A similar genotype-dependent trend was also observed in the brain-to-blood concentration ratio (*P* = 0.13) (Fig. 4d). This difference between App^NL-G-F^ and WT mice was not observed with [^18^F]HTzA. Tetrazines do not have a specific biological target in vivo, but Aβ plaques are known to retain certain substances due to altered tissue composition. Therefore, the observed difference may reflect non-specific retention of [^18^F]MeTzA in Aβ-rich brain regions. Importantly, biodistribution, including analysis of skull uptake demonstrated no genotype-dependent differences in tracer distribution (Fig. 4e, Fig. S3), indicating that tetrazine metabolism did not differ between App^NL-G-F^ and WT mice. Moreover, comparable skull radioactivity was observed for [^18^F]HTzA and [^18^F]MeTzA, suggesting a similar extent of defluorination between tracers. Given the potential non-specific in vivo binding of [^18^F]MeTzA and the higher reactivity of HTzs[37], only [^18^F]HTzA was selected for further use in pretargeted PET imaging studies.

### Pharmacokinetics of DOTA-PEG_11_-Tz

DOTA-PEG_11_-Tz exhibited very low brain concentrations (Fig. S4a), and was able to react with TCOs in plasma (Fig. S4b), indicating its suitability for subsequent pretargeted PET studies to quench circulating TCOs without affecting those within the brain.

### Pretargeted PET imaging with [^18^F]HTzA and TCO-Bapi-Fab8D3 reveals Aβ pathology

**Fig. 5:**
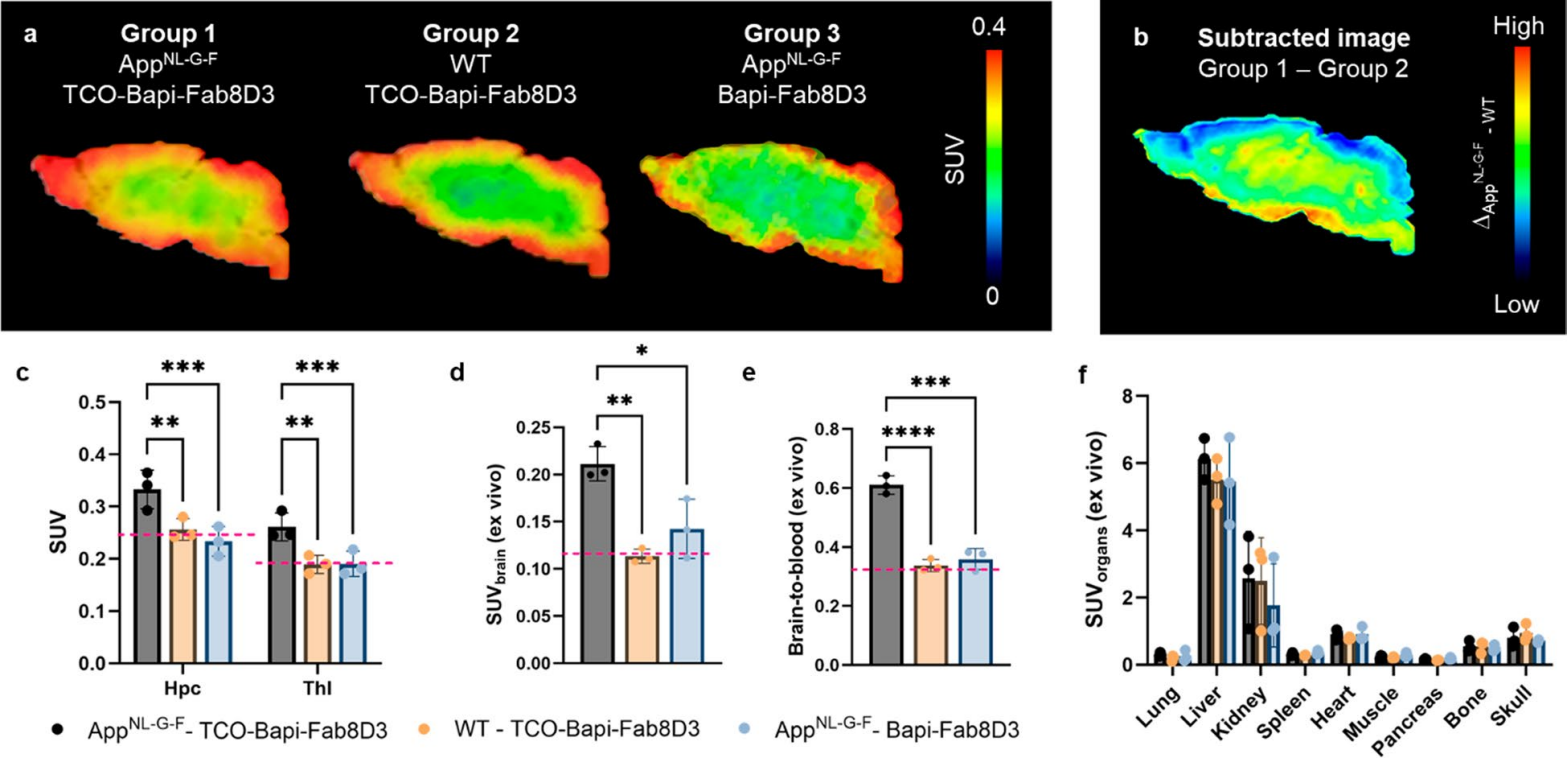
Pretargeted imaging of brain Aβ. **a** Group-averaged sagittal PET images based on activity measured at 3.5–4.5 h post [^18^F]HTzA administration in App^NL-G-F^ and WT mice that had received TCO-Bapi-Fab8D3, and in App^NL-G-F^ mice that had received unmodified Bapi-Fab8D3. The most intense signal was obtained in App^NL-G-F^ mice receiving TCO-Bapi-Fab8D3. **b** An image representing the signal difference between App^NL-G-F^ and WT mice that had received TCO-Bapi-Fab8D3 to illustrate the difference between these groups. **c** Brain radioactivity concentration based on PET imaging, expressed as SUV, in the hippocampus (Hpc) and thalamus (Thl) in App^NL-G-F^ and WT mice that had received TCO-Bapi-Fab8D3, and App^NL-G-F^ mice that had received unmodified Bapi-Fab8D3. **d** Brain radioactivity concentration, expressed as SUV, in perfused isolated brain after PET scanning in App^NL-G-F^ and WT mice that had received TCO-Bapi-Fab8D3, and in App^NL-G-F^ mice that had received Bapi-Fab8D3. **e** Ex vivo brain-to-blood concentration ratio after PET scanning in App^NL-G-F^ and WT mice that had received TCO-Bapi-Fab8D3, and in App^NL-G-F^ mice that had received Bapi-Fab8D3. **f** Radioactivity concentrations in peripheral organs, expressed as SUV, after PET scanning in App^NL-G-F^ and WT mice that had received TCO-Bapi-Fab8D3, and in App^NL-G-F^ mice that had received Bapi-Fab8D3. Average images based on *n* = 3 per group (**a**); difference between average images of App^NL-G-F^ and WT mice (**b**); Mean ± SD of *n* = 3 per condition (**c–e**). The red lines in **c–e** represent concentrations of [^18^F]HTzA in antibody naïve mice. *P < 0.05, **P < 0.01, ***P < 0.001, ***P < 0.0001. Two-way ANOVA Sidak’s multiple comparison (**c**), one-way ANOVA Tukey’s multiple comparison (**d, e**). SUV, standardized uptake value

App^NL-G-F^ and WT mice were administered with TCO-Bapi-Fab8D3 containing an average of 9 TCOs per antibody. As a control, a group of App^NL-G-F^ mice received unmodified Bapi-Fab8D3. PET imaging three days later at 3.5–4.5 h post [^18^F]HTzA administration revealed that the App^NL-G-F^ mice that had received TCO-Bapi-Fab8D3 showed a higher brain signal compared to both WT mice injected with TCO-Bapi-Fab8D3 and App^NL-G-F^ mice that received only the unmodified antibody (Fig. 5a). Both App^NL-G-F^ and WT mice administered with TCO-Bapi-Fab8D3 displayed an elevated signal in the outer regions of the brain, indicating that [^18^F]HTzA reacted with TCOs in these areas, resulting in some spill-in to the brain parenchyma. To visualize differences in brain concentrations between App^NL-G-F^ and WT mice, excluding the contribution from this peripheral spill-in, a subtraction image was generated to isolate the signal originating specifically from Aβ targeting (Fig. 5b). Brain uptake was further quantified in AD-relevant deep brain regions (hippocampus and thalamus) using PET (Fig. 5c), in the whole brain via ex vivo γ-counting of perfused and isolated brain tissue (Fig. 5d), and in relation to terminal [^18^F]HTzA blood concentrations (Fig. 5e). We additionally assessed systemic biodistribution (Fig. 5f), which confirmed that the main peripheral accumulation occurred in the liver followed by the kidneys. No increased retention was observed in spleen, which has a high TfR expression, suggesting that the rate of click reaction between the tetrazine and the TCO-antibody in peripheral TfR-expressing tissues is limited. Across all outcome measures, the App^NL-G-F^ mice that had received TCO-Bapi-Fab8D3 retained more [^18^F]HTzA than both WT mice (30% more in the hippocampus, 38% in the thalamus, 86% in whole brain ex vivo, and 81% in the brain-to-blood ratio) and App^NL-G-F^ mice that received unmodified Bapi-Fab8D3 (43% more in the hippocampus, 37% in the thalamus, 48% in whole brain ex vivo, and 64% in the brain-to-blood ratio). Furthermore, no significant differences were observed between WT mice administered with TCO-Bapi-Fab8D3 (lacking Aβ pathology) and App^NL-G-F^ mice that received unmodified Bapi-Fab8D3 (lacking the TCO handle). In addition, the radioactivity in these mice was very similar to that seen in antibody naïve mice, indicating similar results for all the control mice (Fig. 4). Taken together, these results demonstrate the successful in vivo PET imaging of Aβ pathology using the pretargeted approach.

### Confirmation of Aβ pathology

The presence of Aβ was confirmed in all App^NL-G-F^ mice used in the pretargeted PET study. Aβ levels did not differ between animals that received TCO-Bapi-Fab8D3 and those that received unmodified Bapi-Fab8D3 (Fig. S5).

## Discussion

PET imaging of the brain using a pretargeted approach has been proposed as a promising strategy to enable the use of antibody-based radioligands labeled with short-lived, clinically relevant radionuclides such as ^18^F [16–18, 20]. The use of antibodies instead of small molecule-based radioligands would enable the visualization and quantification of intrabrain protein targets for which no radioligands are currently available, thereby unlocking transformative potential for molecular imaging in neurology.

Despite its promise, pretargeted PET imaging of brain targets has not been successfully implemented until now. One major challenge lies in the need to combine highly brain-penetrant antibodies with tetrazines that possess suitable pharmacokinetic properties; specifically, rapid brain entry and clearance. Without this fine-tuned balance, sufficient imaging contrast cannot be achieved [38]. A recent study demonstrated an alternative version of the pretargeted approach using intrathecally delivered antisense oligonucleotides (ASOs) modified with tetrazine, followed by PET imaging with an ^18^F-labeled TCO [18]. While elegant in concept, this strategy involves an invasive procedure, with ASO-tetrazine delivered via the cisterna magna. In contrast, delivering the pretargeting agent across the BBB provides a more physiologically relevant and less invasive method to reach targets throughout the entire brain parenchyma. Another recent study, currently available only as a preprint, employed a strategy similar to the one presented here [39]. It used a bispecific antibody (biAb) targeting both Aβ and the TfR, and reported increased brain retention of an ^18^F-labeled tetrazine in TCO-biAb-treated AD model mice compared to similarly treated WT mice. However, that study lacked key control groups, specifically, Aβ-expressing mice that were not injected with antibody and mice treated with the unmodified biAb, making it difficult to assess the specificity of the tetrazine retention. Our results underscore the importance of including such controls, as retention of radiolabeled tetrazines in Aβ-rich tissue can occur independently of specific TCO conjugation, potentially confounding the interpretation of imaging specificity. In addition, by including tetrazine-only (no antibody administration) and unmodified antibody controls, and by demonstrating comparable skull uptake across genotypes, we confirm that neither non-specific tetrazine accumulation nor defluorination significantly biases the subtraction-based readout.

The bispecific antibody format used in the present study is similar to the Trontinemab antibody currently being evaluated in clinical trials by Roche. This format, which features monovalent binding to TfR, has been shown to mediate highly efficient BBB transcytosis and high accumulation at Aβ deposits in the brain [28, 29]. These two characteristics are likely the most important features of bispecific antibodies intended for use in a pretargeted imaging approach. We note that although TfR-mediated shuttling increases brain delivery, absolute brain uptake remains modest relative to systemic exposure. Nevertheless, the uptake achieved is sufficient for robust Aβ-specific PET imaging, and the low accumulation in off-target TfR-expressing organs, such as the spleen, further supports the safety of this approach. Peripheral accumulation was primarily observed in the liver and kidneys, consistent with normal clearance pathways, whereas no increased splenic retention was detected in the pretargeting experiments, likely reflecting limited formation of clicked product in peripheral TfR-expressing tissues.

In addition to the Roche-format, we have previously used other bispecific antibody formats labeled with iodine-124 for direct PET imaging of brain Aβ [11, 12, 35, 40–42]. In those studies, the difference in radioligand uptake between AD and WT mice was somewhat more pronounced than that observed here. These observations suggest that optimizing TCO load on the antibody could further improve the contrast in pretargeted imaging. However, it is essential to ensure that antibody affinity for both Aβ and TfR is maintained if higher TCO loading is applied. Previous studies have shown that excessive TCO conjugation can reduce antibody affinity and impair transcytosis across the BBB via TfR-mediated delivery. Therefore, careful optimization of TCO modification remains a key factor in maximizing imaging performance. In addition, further improvements could be achieved by refining other components of the pretargeting system. For instance, developing ^18^F-tetrazines with enhanced brain delivery and rapid brain washout of unbound tetrazine could improve the target-to-background ratio. In our study, we compared two tetrazine variants: the more lipophilic [^18^F]MeTzA and the more hydrophilic [^18^F]HTzA. While [^18^F]MeTzA initially demonstrated higher brain uptake, it accumulated to a greater extent in App^NL-G-F^ mice compared to WT controls. This suggests that its lipophilicity may contribute to non-specific retention in Aβ-rich regions.

We evaluated kinetics of both tetrazines at 1.5 and 4 h post-injection and observed similar brain concentrations at the later time-point across genotypes, accompanied by increased washout after 4 h relative to 1.5 h. This supports 4 h post-injection as a suitable imaging time for maximizing contrast in the current system.

Considering these findings and the higher reactivity of [^18^F]HTzA, we selected [^18^F]HTzA for use in the final pretargeted imaging experiments. Nevertheless, a slightly more lipophilic [^18^F]HTzA derivative with balanced properties could further improve the approach [17, 37]. In future work, the development of tetrazines with even faster systemic and brain clearance may enable earlier imaging time-points while maintaining high specificity.

Similarly, optimizing antibody dose and systemic clearance, either through antibody engineering or by improving the efficiency of the blocking or clearing agents, may contribute to reducing off-target binding and enhancing imaging contrast. Together, these considerations highlight both the promise of pretargeted immunoPET and the importance of continued optimization of shuttle affinity, tetrazine pharmacokinetics, and TCO loading to support translation.

## Conclusions

In this study, we demonstrate successful pretargeted PET imaging of brain Aβ pathology using a bispecific antibody engineered for TfR-mediated transport and modified with TCO. This approach enabled the use of ^18^F-labeled tetrazines for high-contrast imaging, overcoming limitations of poor BBB penetration and reliance on long-lived radionuclides. While the current work focused on Aβ, the strategy is broadly applicable to other brain targets, provided a suitable antibody is available. Further optimization of tetrazine properties and TCO loading may enhance imaging performance. Overall, this pretargeted PET methodology represents a major advance in molecular imaging, with potential for non-invasive visualization of previously inaccessible brain proteins in both research and clinical settings.

## Supplementary Information

Additional file 1. **Fig. S1**. Chemical structures of tetrazines used. **Fig. S2**. Brain PET concentrations at 1.5 hours and 4 hours post tracer administration of two ¹⁸F-tetrazines. **Fig. S3**. Ex vivo skull retention at 5 hours post administration of [^18^F]HTzA and [^18^F]MeTzA in App^NL-G-F^ and WT mice. **Fig. S4**. Brain concentrations of [⁶⁸Ga]Ga-DOTA-PEG₁₁-Tz. **Fig. S5**. Amyloid-β (Aβ) levels in the brains of mice included in the pretargeted PET study. **Table S1**. Mice used in the different studies.

Additional file 2: Uncropped radioTLC.

## Data Availability

All data are available in the main text or the supplementary materials. The datasets used and/or analyzed during the current study are available from the corresponding author on reasonable request.

## Abbreviations

^1^^8^F: Fluorine-18 (radionuclide)
^68^ Ga: Gallium-68 (radionuclide)
AD: Alzheimer’s disease
Aβ: Amyloid-β
A_m_: Molar activity
BBB: Blood–brain barrier
CT: Computed tomography
DMSO: Dimethylsulfoxide
ELISA: Enzyme-linked immunosorbent assays
hIgG: Human immunoglobulin G
i.p: Intraperitoneal
i.v.: Intravenous
LCO: Luminescent Conjugated Oligothiophene
PET: Positron emission tomography
Radio-TLC: Radio-thin layered chromatography
RCY: Radiochemical yield
RCP: Radiochemical purity
SPE: Solid-phase extraction
TCO: Trans-cyclooctene
TfR: Transferrin receptor
SUV: Standardized uptake value
VOI: Volume of interest
