# Supplementary material for: Pretargeted brain PET imaging reveals amyloid-β pathology using a TCO-modified antibody and a fluorine-18-labeled tetrazine
Source: Transl Neurodegener. 2025 Dec 26;14:72. doi: 10.1186/s40035-025-00532-2 (PMC12742193; doi:10.1186/s40035-025-00532-2)
Supplement: Supplementary file 1 — Additional file 1. Fig. S1. Chemical structures of tetrazines used. Fig. S2. Brain PET concentrations at 1.5 hours and 4 hours post tracer administration of two ¹⁸F-tetrazines. Fig. S3. Ex vivo skull retention at 5 hours post administration of [18F]HTzA and [18F]MeTzA in AppNL-G-F and WT mice. Fig. S4. Brain concentrations of [⁶⁸Ga]Ga-DOTA-PEG₁₁-Tz. Fig. S5. Amyloid-β (Aβ) levels in the brains of mice included in the pretargeted PET study. Table S1. Mice used in the different studies. [file 40035_2025_532_MOESM1_ESM.docx]

**Supplementary Materials**

**Fig. S1. Chemical structures of tetrazines used.** A polar gallium-68 (^68^Ga)radiolabeled tetrazine, [^68^Ga]-DOTA-PEG_11_-Tz, was used for ex vivo experiments, while two fluorine-18 (¹⁸F) radiolabeled tetrazines, [^18^F]HTzA and [^18^F]MeTzA, were evaluated in vivo.


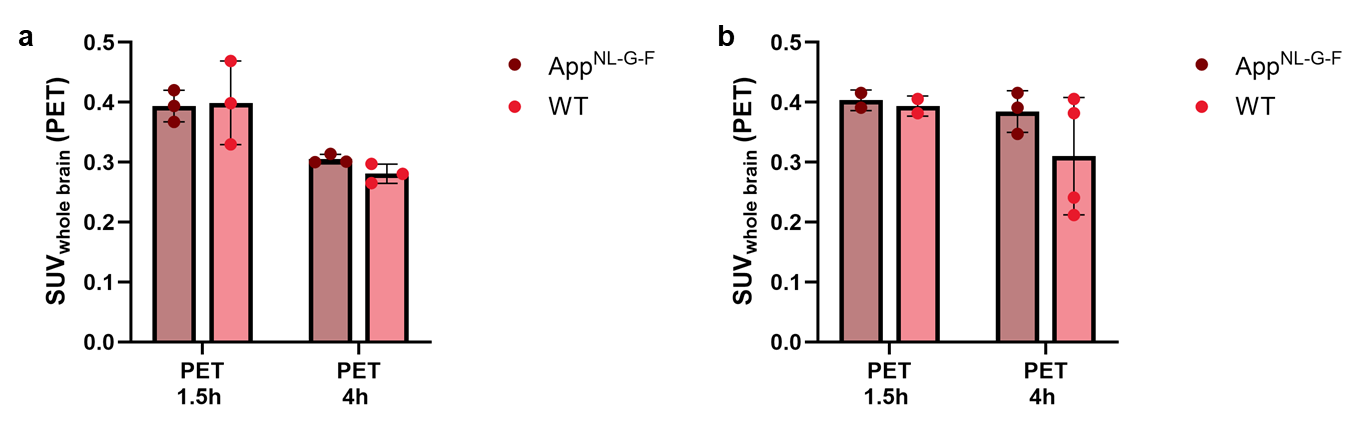


**Fig. S2. Brain PET concentrations at 1.5 hours and 4 hours post tracer administration of two ¹⁸F-tetrazines. (a)** [^18^F]HTzA **(b)** [^18^F]MeTzA, expressed as SUV, in App^NL-G-F^ and WT mice.


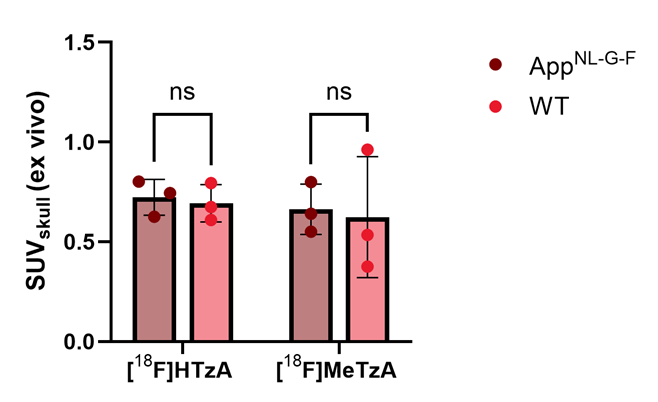


**Fig. S3. Ex vivo skull retention at 5 hours post administration of [^18^F]HTzA and [^18^F]MeTzA in App^NL-G-F^ and WT mice.** Mean ± SD of *n* = 3 for HTzA per genotype and *n* = 3 for MeTzA per genotype; mean ± SD of *n* = 3 per condition. ns: *P* > 0.05. Two-way ANOVA Sidak’s multiple comparison. SUV=standardized uptake value, a dose and weight normalized concentration.


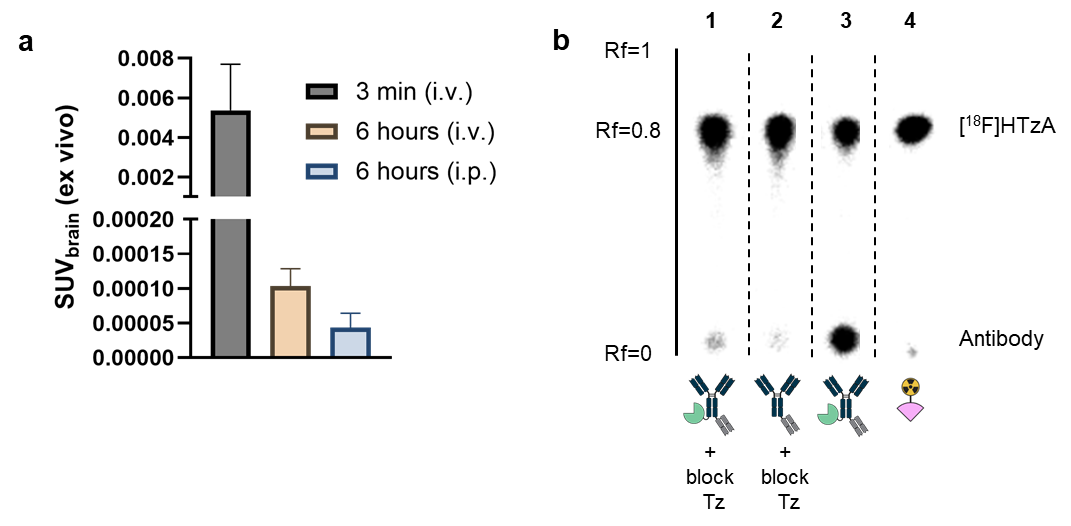


**Fig. S4. Brain concentrations of [⁶⁸Ga]Ga-DOTA-PEG₁₁-Tz. a** A polar tetrazine, radiolabeled with gallium-68 ([⁶⁸Ga]Ga-DOTA-PEG₁₁-Tz), was either intravenously or intraperionally administered to WT mice to ensure that the tetrazine did not enter the brain. The resulting brain concentrations were 100- to 1000-fold lower than those seen with the fluorine-18 (¹⁸F)-labeled brain-penetrant tetrazines used for pretargeting. **b** Representative radioTLC data. Plasma samples were collected from mice receiving: (1) TCO-Bapi8D3-Fab8D3 and DOTA-PEG₁₁-Tz (blocking tetrazine); (2) Bapi8D3-Fab8D3 and DOTA-PEG₁₁-Tz (blocking tetrazine); (3) Bapi8D3-Fab8D3 without DOTA-PEG₁₁-Tz (no blocking); and (4) PBS as a control. All samples were incubated with [¹⁸F]HTzA for 30 minutes at 37 °C and then applied to NP silica TLC plates. The mobile phase consisted of 8:2 ethyl acetate/n-heptane. Radioactivity distribution was visualized using a Cyclone phosphor imaging system. These data show that pre-injection of DOTA-PEG₁₁-Tz effectively reduced [¹⁸F]HTzA binding to circulating TCO-labeled antibody, whereas in the absence of blocking, [¹⁸F]HTzA successfully underwent click ligation to the antibody in plasma. Mean ± SD of *n* = 3 per condition (**a**). (TLC = Thin-Layer Chromatography). Uncropped radioTLC is shown in Supplementary Material Files 2 and 3.

**
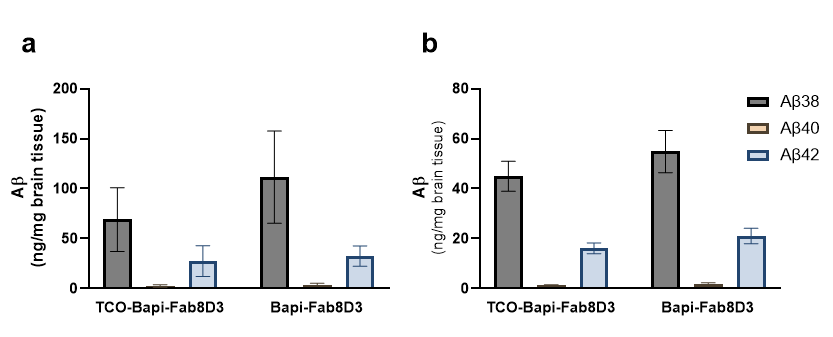
**

**Fig. S5. Amyloid-β (Aβ) levels in the brains of mice included in the pretargeted PET study. a** Aβ levels in the cerebrum of App^NL-G-F^ mice that received either TCO-Bapi-Fab8D3 or Bapi-Fab8D3 prior to pretargeted PET with [^18^F]HTzA. **b** Aβ levels in the cerebellum of App^NL-G-F^ mice that received either TCO-Bapi-Fab8D3 or Bapi-Fab8D3 prior to pretargeted PET with [^18^F]HTzA. There were no differences between the two groups of mice for any of the Aβ species. Mean ± SD of *n* = 3 per condition. Two-way ANOVA Sidak’s multiple comparison.

**Table S1. Mice used in the different studies**

| Study | Number of AD mice (m/f) | Number of WT | Age (months) |
| --- | --- | --- | --- |
| Ex vivo pretargeting | 6 (2/4) | 3 (0/3) | 18-19 |
| Selection of [^18^F]Tz | 6 (1/5) | 7 (1/6) | 12-17 |
| Ex vivo [^68^Ga]Tz | n.a. | 9 (5/4) | 4 |
| PET Pretargeting | 6 (3/3) | 3 (2/1) | 16-19 |
