# Supplementary figures and images for: Pretargeted brain PET imaging reveals amyloid-β pathology using a TCO-modified antibody and a fluorine-18-labeled tetrazine
Source: Transl Neurodegener. 2025 Dec 26;14:72. doi: 10.1186/s40035-025-00532-2 (PMC12742193; doi:10.1186/s40035-025-00532-2)

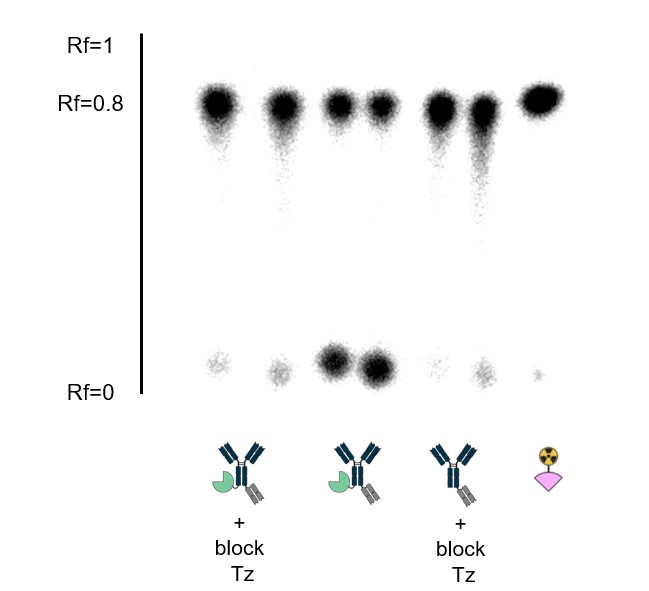

Supplement: Supplementary file 2 — Additional file 2: Uncropped radioTLC. [file 40035_2025_532_MOESM2_ESM.jpg]
